# Identification of candidate genes and proteins in aging skeletal muscle (sarcopenia) using gene expression and structural analysis

**DOI:** 10.7717/peerj.5239

**Published:** 2018-09-05

**Authors:** Gita Shafiee, Yazdan Asgari, Akbar Soltani, Bagher Larijani, Ramin Heshmat

**Affiliations:** 1 Chronic Diseases Research Center, Endocrinology and Metabolism Population Sciences Institute, Tehran University of Medical Sciences, Tehran, Iran; 2 Department of Medical Biotechnology, School of Advanced Technologies in Medicine, Tehran University of Medical Sciences, Tehran, Iran; 3 Endocrinology and Metabolism Research Center, Endocrinology and Metabolism Clinical Sciences Institute, Tehran University of Medical Sciences, Tehran, Iran

**Keywords:** Skeletal muscle, Structural analysis, Sarcopenia, Aging

## Abstract

Sarcopenia is an age-related disease characterized by the loss of muscle mass and muscle function. A proper understanding of its pathogenesis and mechanisms may lead to new strategies for diagnosis and treatment of the disease. This study aims to discover the underlying genes, proteins, and pathways associated with sarcopenia in both genders. Integrated analysis of microarray datasets has been performed to identify differentially expressed genes (DEGs) between old and young skeletal muscles. Gene Ontology (GO) enrichment analysis and Kyoto Encyclopedia of Genes and Genomes (KEGG) pathway enrichment analysis were then performed to uncover the functions of the DEGs. Moreover, a protein–protein interaction (PPI) network was constructed based on the DEGs. We have identified 41,715 DEGs, including 19 downregulated and 41,696 upregulated ones, in men. Among women, 3,015 DEGs have been found, with 2,874 of them being upregulated and 141 downregulated genes. Among the top up-regulated and downregulated genes, the ribosome biogenesis genes and genes involved in lipid storage may be closely related to aging muscles in men and women respectively. Also, the DEGs were enriched in the pathways including those of ribosome and Peroxisome proliferator-activated receptor (PPAR) in men and women, respectively. In the PPI network, Neurotrophic Receptor Tyrosine Kinase 1 (NTRK1), Cullin 3 (CUL3) and P53 have been identified as significant hub proteins in both genders. Using the integrated analysis of multiple gene expression profiles, we propose that the ribosome biogenesis genes and those involved in lipid storage would be promising markers for sarcopenia in men and women, respectively. In the reconstructed PPI network, neurotrophic factors expressed in skeletal muscle are essential for motoneuron survival and muscle fiber innervation during development. Cullin E3 ubiquitin ligase (Cul3) is an important component of the ubiquitin–proteasome system—it regulates the proteolysis. P53 is recognized as a central regulator of the cell cycle and apoptosis. These proteins, which have been identified as the most significant hubs, may be involved in aging muscle and sarcopenia.

## Introduction

Sarcopenia is an age-related disease characterized by the loss of muscle mass and muscle function, and it leads to an increased risk of adverse outcomes such as falling, functional limitation, disability, and mortality in elderly people (Cruz-Jentoft et al., 2010; Houston, Nicklas & Zizza, 2009).

The age-associated reduction in muscle mass, the main parameter of sarcopenia, is complex as it involves multi-biological processes. Although there are multiple types of molecular pathology, including mitochondrial dysfunction, insulin resistance, inflammatory states and loss of *α*-motor, and biological changes, that participate in the development of the aging of muscle mass (Petersen et al., 2003; Walston, 2012), the primary cause of sarcopenia is unclear. Also, these changes in muscle mass are regulated through different mechanisms in men and women (Iannuzzi-Sucich, Prestwood & Kenny, 2002).

A better understanding of molecular pathology would provide a greater insight into the aging process of muscle mass and could be beneficial to early diagnosis and prevention of aging-related diseases such as sarcopenia. Recently, the use of microarrays as a powerful technique has been utilized to identify a global view of the molecular changes in various states. In previous studies, this tool has been used to research the impact of aging on muscle mass (Liu et al., 2010; Welle, Tawil & Thornton, 2008). Also, to identify the molecular mechanisms of sarcopenia, several studies have been performed based on the gene expression of muscle mass during aging by microarray technology (Bortoli et al., 2003; Giresi et al., 2005; Zahn et al., 2006). Although these analyses of muscle biopsies have been used to detect new genes and pathways associated with muscle function, there are still some limitations relating to this approach. The different sample sources, array platforms, and analysis techniques may make it difficult to compare between different studies. Therefore, it seems essential to integrate different gene expression datasets derived from various microarray studies of the muscle aging process to overcome the limitations of individual datasets. This would resolve inconsistencies as well.

The current study aims to integrate gene expression profiles for identifying the differentially expressed genes (DEGs) of young and old skeletal muscle samples in both genders as well as to find hub genes. In addition, a proper understanding of the PPI interaction and hub proteins may provide some insights into further exploration of the pathogenic mechanism of sarcopenia.

## Material and Methods

### Data attainment and preprocessing

The gene expression data of sarcopenia has been obtained from the Gene Expression Omnibus (GEO; http://www.ncbi.nlm.nih.gov/geo/) (Barrett et al., 2013). The following search terms were used: “sarcopenia,” “muscle,” and “age.”

The studies included in this study compared gene expression profiles between the muscle mass of both old and young in both genders. Non-human studies and reviews were excluded. Applying the above-mentioned conditions, two types of gene expression data were selected—GSE38718 (Liu et al., 2013) and GSE25941 (Raue et al., 2012)—which were performed based on the GPL570 Affymetrix Human Genome U 133 plus 2.0 Array platform (Table 1).

**Table 1:** Characteristics of the individual studies.

GEO ID	Male/Female	Young/Old	Platform	Sample source	Country	Year	Ref.
GSE38718	21/21	22/20	GPL570 Affymetrix Human Genome	Muscle (Biceps brachii)	USA	2013	Chen et al. (2009)
GSE25941	17/19	15/21	GPL570 Affymetrix Human Genome	Muscle (Vastuslateralis)	USA	2012	Chin et al. (2014)

We used the MATLAB software (R2014a) to analyze the chip data. For the preprocessing step, we performed the log2 transformation and then applied the quantile normalization process to raw data to minimize the heterogeneity among microarray studies from different samples. In the next step, we chose a *p*-value less than 0.05 and log fold change (FC) >2.0 as significant thresholds to obtain differentially expressed genes (DEGs) between old and young muscle mass groups for both men and women.

### Detection of hub genes

We used the Cytoscape software (version 3.2.1) for reconstructing the networks and structural analysis. We performed two different approaches for the structural analysis and function prediction: First, a Cytoscape plugin called GeneMANIA was used for building a gene–gene interaction network from a list of DEGs (http://www.genemania.org/plugin/) (Montojo et al., 2010) as well as for analyzing the constructed network to provide gene function prediction. The hub genes of the network were detected using the cytoHubba plugin for Cytoscape and the node degree was chosen as a centrality index. (Chin et al., 2014). A node degree is explained with the number of neighbors connected directly to the node, while the nodes with higher degrees are considered as hubs.

Next, a protein–protein interaction (PPI) network analysis was performed as another structural approach to explore the functions of the DEGs. The BisoGenet plugin of Cytoscape (https://omictools.com/bisogenet-tool) was used to construct a PPI network based on the list of the DEGs (Martin et al., 2010). Next, a centrality analysis using the cytoHubba plugin was performed to explore hub proteins with high node degrees as a centrality index.

### Pathway enrichment analysis of DEGs

We used two different online resources, ToppGene (https://toppgene.cchmc.org/) (Chen et al., 2009) and InnateDB (http://www.innatedb.com/) (Breuer et al., 2013), to detect the biological functions and potential pathways of the DEGs. These websites perform function prediction based on gene ontology (GO) and KEGG enrichment analysis approaches. Enriched GO terms/KEGG pathways were selected based on a false discovery rate (FDR) <0.01 as a threshold.

## Results

By the integrated analysis, we identified 41,715 DEGs, including 19 downregulated and 41,696 upregulated DEGs, in men. Among women, 3,015 DEGs were found with 2,874 upregulated and 141 downregulated genes. The full list of the DEGs has been provided in Table S1. To identify potential hub genes, the node degree was considered as a centrality index in both upregulated and downregulated genes for both genders. The top hub genes for both genders have been demonstrated in Table 2 and Table S2 .

**Table 2:** Top hub nodes based on the GeneMANIA reconstructed network.

Men			Women		
Gene symbol	Gene name	Degree	Gene symbol	Gene name	Degree
RPL24	*Ribosomal Protein L24*	117	PLIN1	*Perilipin 1*	39
RPS3	*Ribosomal Protein S3*	108	FABP4	*Fatty Acid Binding Protein 4*	39
RPL6	*Ribosomal Protein L6*	107	TIMP4	*Tissue Inhibitor Of Metalloproteinase 4*	35
RPS5	*Ribosomal Protein S5*	106	ADH1B	*Alcohol Dehydrogenase 1B*	34
PSMA3	*Proteasome Subunit Alpha3*	105	GPC3	*Glypican 3*	33
RPL11	*Ribosomal Protein L11*	103	PRL	*Prolactin*	32
RPS16	*Ribosomal Protein S16*	101	LPL	*Lipoprotein Lipase*	31
PSMA1	*Proteasome Subunit Alpha1*	98	G0S2	*G0/G1 Switch 2*	30
RPL12	*Ribosomal Protein L12*	98	PCK1	*Phosphoenolpyruvate Carboxykinase 1*	30
PSMB1	*Proteasome Subunit Beta1*	96	GYG2	*Glycogenin 2*	29
PSMA4	*Proteasome Subunit Alpha4*	96	GPD1	*Glycerol-3-Phosphate Dehydrogenase 1*	29
RPL27	*Ribosomal Protein L27*	94	ADIPOQ	*Adiponectin*	29
SERPINE2	*Serpin Family Member E2*	12	MNDA	*Myeloid Cell Nuclear Differentiation Antigen*	32
SERPINB2	*Serpin Family Member B2*	12	S100A8	*S100 Calcium Binding Protein A8*	30
SERPINE1	*Serpin Family Member E 1*	11	ITGB6	*Integrin Subunit Beta 6*	27
ZMIZ1	*Zinc Finger MIZ-Type Containing 1*	11	PROK2	*Prokineticin 2*	26
SERPINB4	*Serpin Family Member B4*	11	S100A9	*S100 Calcium Binding Protein A9*	25
SERPINB3	*Serpin Family Member B3*	10	ADCYAP1	*Adenylate Cyclase Activating Polypeptide 1*	23
PIAS2	*Protein Inhibitor Of Activated STAT 2*	10	S100A12	*S100 Calcium Binding Protein A12*	23
SERPINF1	*Serpin Family Member F1*	9	RGS6	*Regulator Of G-Protein Signaling 6*	23
SERPINA1	*Serpin Family Member A1*	9	KPNA4	*Karyopherin Subunit Alpha 4*	23
HMSD	*Histocompatibility Minor Serpin Domain Containing*	9	ADCYAP1R1	*ADCYAP Receptor Type I*	22
SERPINE3	*Serpin Family Member E3*	9	DUSP3	*Dual Specificity Phosphatase 3*	22
PIAS4	*Protein Inhibitor Of Activated STAT 4*	8	VEGFA	*Vascular Endothelial Growth Factor A*	22

GO analysis showed the enrichment of DEGs in biological processes, molecular functions, and cellular components. Among men, the enriched GO term for the biological process was the negative regulation of molecular function; the GO term for the cellular component was cytosolic ribosome; and serine-type endopeptidase inhibitor activity was significantly enriched for molecular functions.

We found that the enriched GO terms among women were chemokine production, lipid particle, and RAGE receptor binding for biological processes, molecular functions, and cellular components respectively. The full list of GO term categories enrichment analysis is shown in Tables 3 and 4 in men and women respectively.

Furthermore, to explore the biological significance of the DEGs, the KEGG pathway enrichment analysis was also conducted. Among men, the most significantly enriched pathway was ribosome. Furthermore, the DEGs were significantly enriched in pathway in proteasome and amoebiasis. Among men, the most significantly enriched pathway was ribosome. Moreover, the DEGs were significantly enriched in the pathway in proteasome and amoebiasis. Among women, the peroxisome proliferator-activated receptor (PPAR) signaling pathway and the adipocytokine signaling pathway were highly enriched. Also, the Glycolysis / Gluconeogenesis was found to be significantly enriched (Table 5).

**Table 3:** The enriched gene ontology (GO) categories of differentially expressed genes (DEGs) in men.

GO ID	Terms	Count	*P*-value	FDR
**Biological process**				
GO:0044092	negative regulation of molecular function	16	1.86E−14	1.83E−11
GO:0030162	regulation of proteolysis	14	3.77E−14	1.85E−11
GO:0006614	SRP-dependent cotranslational protein targeting to membrane	8	2.00E−13	6.55E−11
GO:0006613	cotranslational protein targeting to membrane	8	3.61E−13	8.13E−11
GO:0045047	protein targeting to ER	8	4.24E−13	8.13E−11
GO:0052548	regulation of endopeptidase activity	11	5.72E−13	8.13E−11
GO:0072599	establishment of protein localization to endoplasmic reticulum	8	5.79E−13	8.13E−11
GO:0052547	regulation of peptidase activity	11	1.06E−12	1.25E−10
GO:0016032	viral process	13	1.14E−12	1.25E−10
GO:0044764	multi-organism cellular process	13	1.44E−12	1.41E−10
GO:0000184	nuclear-transcribed mRNA catabolic process, nonsense-mediated decay	8	1.58E−12	1.41E−10
GO:0070972	protein localization to endoplasmic reticulum	8	2.20E−12	1.77E−10
GO:0044419	interspecies interaction between organisms	13	2.53E−12	1.77E−10
GO:0044403	symbiosis, encompassing mutualism through parasitism	13	2.53E−12	1.77E−10
GO:0032269	negative regulation of cellular protein metabolic process	14	7.06E−12	4.55E−10
GO:0043086	negative regulation of catalytic activity	13	7.41E−12	4.55E−10
**Cellular component**				
GO:0022626	cytosolic ribosome	8	1.18E−12	1.14E−10
GO:0044391	ribosomal subunit	8	2.67E−11	1.29E−09
GO:0005840	ribosome	8	2.81E−10	6.81E−09
GO:0044445	cytosolic part	8	2.81E−10	6.81E−09
GO:0005839	proteasome core complex	4	9.03E−09	1.32E−07
GO:0030529	intracellular ribonucleoprotein complex	10	9.55E−09	1.32E−07
GO:1990904	ribonucleoprotein complex	10	9.55E−09	1.32E−07
GO:0022625	cytosolic large ribosomal subunit	5	1.81E−08	2.19E−07
GO:0019773	proteasome core complex, alpha-subunit complex	3	9.61E−08	1.04E−06
GO:0015934	large ribosomal subunit	5	1.75E−07	1.70E−06
GO:0000502	proteasome complex	4	2.53E−06	2.23E−05
GO:0005925	focal adhesion	6	7.03E−06	5.62E−05
GO:0005924	cell-substrate adherens junction	6	7.56E−06	5.62E−05
GO:0030055	cell-substrate junction	6	8.12E−06	5.62E−05
**Molecular function**				
GO:0004867	serine-type endopeptidase inhibitor activity	9	1.96E−15	2.05E−13
GO:0004866	endopeptidase inhibitor activity	9	3.97E−13	1.66E−11
GO:0061135	endopeptidase regulator activity	9	5.44E−13	1.66E−11
GO:0030414	peptidase inhibitor activity	9	6.33E−13	1.66E−11
GO:0061134	peptidase regulator activity	9	4.39E−12	9.22E−11
GO:0003735	structural constituent of ribosome	8	1.66E−10	2.91E−09
GO:0004857	enzyme inhibitor activity	9	6.83E−10	1.03E−08
GO:0004298	threonine-type endopeptidase activity	4	1.20E−08	1.40E−07
GO:0070003	threonine-type peptidase activity	4	1.20E−08	1.40E−07
GO:0019843	rRNA binding	5	1.98E−08	2.08E−07
GO:0030234	enzyme regulator activity	9	2.55E−06	2.44E−05
GO:0005198	structural molecule activity	8	2.88E−06	2.52E−05

**Table 4:** The enriched gene ontology (GO) categories of differentially expressed genes (DEGs) in women.

GO ID	Terms	Count	*P*-value	FDR
**Biological process**				
GO:0032602	chemokine production	5	7.97E−08	1.01E−04
GO:0006954	inflammatory response	9	1.23E−07	1.01E−04
GO:0050727	regulation of inflammatory response	7	1.75E−07	1.01E−04
GO:0048878	chemical homeostasis	10	5.74E−07	2.17E−04
GO:0050729	positive regulation of inflammatory response	5	6.29E−07	2.17E−04
GO:1903034	regulation of response to wounding	7	1.53E−06	4.40E−04
GO:0032103	positive regulation of response to external stimulus	6	2.84E−06	7.02E−04
GO:1903036	positive regulation of response to wounding	5	3.56E−06	7.68E−04
GO:0070488	neutrophil aggregation	2	4.69E−06	9.00E−04
GO:0009967	positive regulation of signal transduction	10	6.59E−06	1.14E−03
GO:0016051	carbohydrate biosynthetic process	5	7.46E−06	1.17E−03
**Cellular component**				
GO:0005811	lipid particle	3	7.83E−05	6.97E−03
GO:0005615	extracellular space	8	2.60E−04	1.16E−02
**Molecular function**				
GO:0050786	RAGE receptor binding	3	3.98E−07	6.25E−05
GO:0035662	Toll-like receptor 4 binding	2	9.34E−06	4.04E−04
GO:0031406	carboxylic acid binding	5	1.13E−05	4.04E−04
GO:0043177	organic acid binding	5	1.20E−05	4.04E−04
GO:0005102	receptor binding	10	1.29E−05	4.04E−04
GO:0005504	fatty acid binding	3	2.85E−05	7.31E−04
GO:0050544	arachidonic acid binding	2	3.26E−05	7.31E−04
GO:0050543	icosatetraenoic acid binding	2	4.34E−05	7.97E−04
GO:0050542	icosanoid binding	2	5.58E−05	7.97E−04
GO:0035325	Toll-like receptor binding	2	5.58E−05	7.97E−04
GO:1901567	fatty acid derivative binding	2	5.58E−05	7.97E−04

**Table 5:** The significantly enriched KEGG pathways of differentially expressed genes (DEGs) in both genders.

Pathway term	Gene count	*P*-value	FDR
**Men**			
Ribosome	8	9.05E−11	7.40E−10
Proteasome	3	1.00E−04	5.79E−04
Amoebiasis	3	0.001197846	0.001632358
**Women**			
PPAR signaling pathway	5	3.91E−07	2.15E−05
Adipocytokine signaling pathway	2	0.010953677	0.035438366
Glycolysis/Gluconeogenesis	2	0.010069903	0.036922978

**Figure 1:**
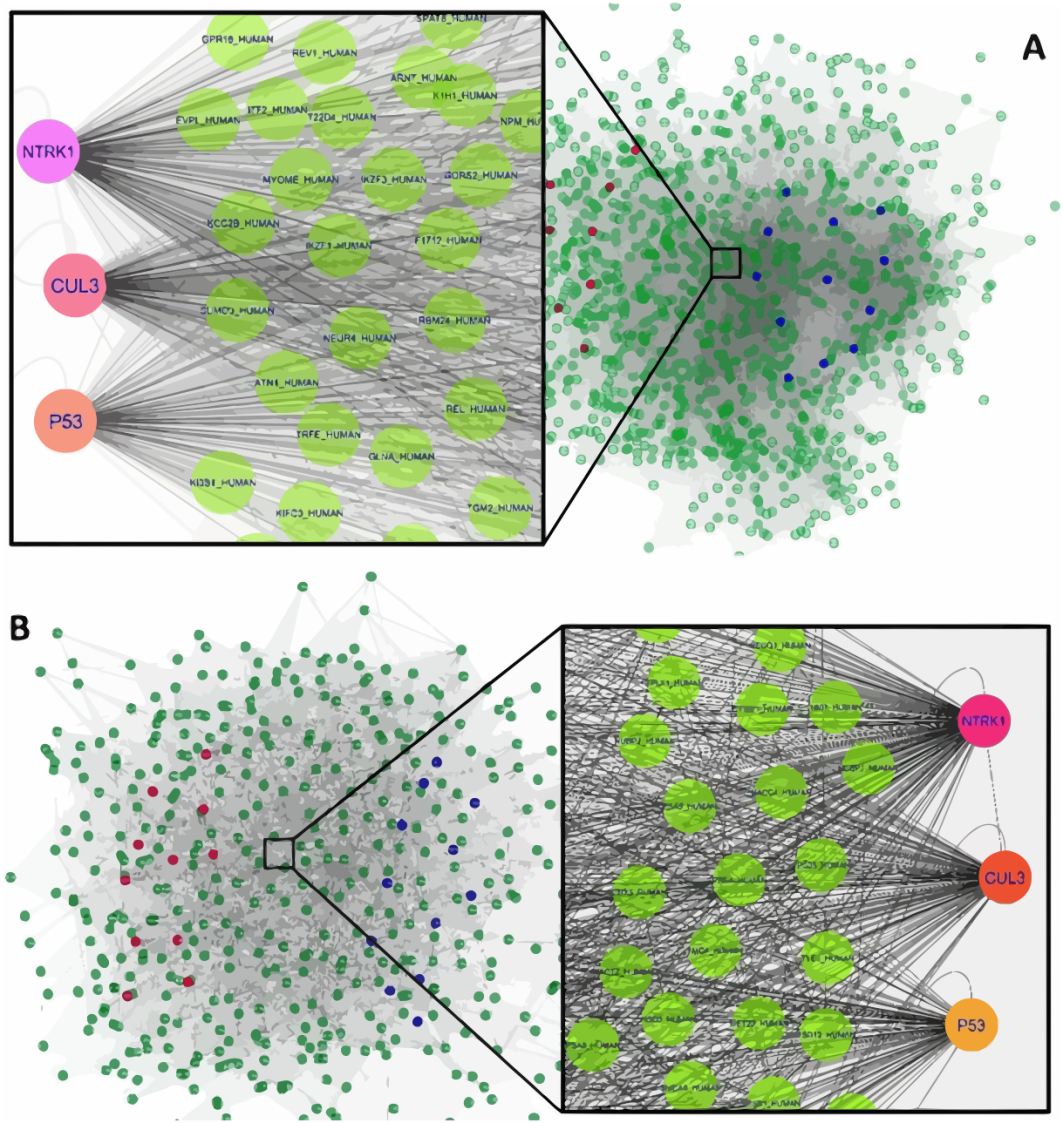
The constructed PPI network of the top up- and down-regulated DEGs in Men (A) and Women (B). Nodes denote proteins; edges denote interactions between two proteins. Red and blue nodes represent products of down- and up-regulated DEGs, respectively.

The interaction network of top upregulated and downregulated DEGs were established by the Cytoscape software. In the PPI network of men, there were 1,189 nodes and 28,982 edges, while 454 nodes and 4,556 edges were in the network of women. Among men, three nodes with the highest degree were defined as hub proteins, including Neurotrophic Receptor Tyrosine Kinase 1 (NTRK1, degree = 409), Cullin 3 (CUL3, degree = 336), and P53 (degree = 276). The significant hub proteins in women contained NTRK1 (degree = 168), CUL3 (degree = 133), and P53 (degree = 104) (Fig. 1).

## Discussion

The molecular mechanisms of skeletal muscle maintenance/development involve an interplay between multiple signaling pathways. In normal conditions, a network of pathways, genes, and interconnected proteins serves to control hypertrophic and atrophic messages with the balance between muscle protein synthesis and proteolysis. The key signaling pathway for muscle protein synthesis is insulin-like growth factor-1- (IGF1), PtdIns-3-OH kinase-(PI3K)-AKt. IGF1- PI3K-AKt signaling promotes skeletal muscle hypertrophy by activating the mammalian target of rapamycin (mTOR). The activation of mTOR in response to growth factors, feeding, and increased mechanical loading is a key step in inducing muscle hypertrophy by increasing protein synthesis. Additionally, Akt inhibits protein degradation through Forkhead box O (FOXO)-mediated proteasome activity (Egerman & Glass, 2014; Hanaoka et al., 2012; Rommel et al., 2001). Proteolytic systems, including calpain, the ubiquitin–proteasome pathway, and the autophagy–lysosomal pathway, are responsible for protein degradation in muscle cells (Purintrapiban, Wang & Forsberg, 2003). The ubiquitin–proteasome system is a key mechanism for the control of metabolic enzymes or dedicated regulatory proteins (Bonaldo & Sandri, 2013).

According to this content, the onset of age-related muscle loss (sarcopenia) is developed by an imbalance between muscle protein synthesis and proteolysis. Sarcopenia is an age-related disease that has rapidly increased around the world. The understanding of the molecular mechanisms for sarcopenia is an important factor to develop better detection, diagnosis, and novel therapeutic targets of this disease. Increased proteolysis and decreased protein synthesis in sarcopenia is attributed to the activity of the ubiquitin–proteasome system interconnected with autophagy.

Most studies on sarcopenia focused on individual genes and lacked global data. Given that the main pathways interact with other mechanisms, an integrated look could be considered as an essential aim to identify hub genes and proteins–it may improve the clinical management of disease. In this study, we focused on the DEGs between old and young muscle mass based on two microarray studies to identify the key genes, pathways, and hub proteins involved in the development of low muscle mass as the main parameter of sarcopenia.

In total, 41,715 genes were filtered as the DEGs with 41,696 upregulated and 19 downregulated genes in men. Among women, we identified 3,015 DEGs, which included 2,874 upregulated and 141 downregulated genes.

Some evidence indicated the importance of differences in gene expressions in explaining muscle development and muscle function between men and women (Eason et al., 2000; Rosenkranz-Weiss et al., 1994; Te Pas et al., 1999).

However, the basis for these sex-related differences is not clear: estrogen and androgen elements might influence the sex-related differences in gene expressions (Roth et al., 2002). It seems that some effects of testosterone on gene expressions might be limited to the period of rapid muscle growth after puberty (Welle, Tawil & Thornton, 2008). The significant differences in body fat proportions between men and women are another possibility of causing these differences in gene expressions (Roth et al., 2002).

Some evidence was collected for muscle gene expression profiles in men and women (Roth et al., 2002; Welle et al., 2004; Welle et al., 2003). Welle, Tawil & Thornton (2008) reported that sex-related differences in muscle expressions, such as genes coding mitochondrial proteins and ribosomal biogenesis, could contribute to differences in the muscle size of men and women. Another study found that sex had the strongest influence on muscle gene expressions, with differential expressions (>1.7-fold) being observed in this regard (Roth et al., 2002). In addition, many age-related changes in muscle mass, such as insulin resistance, pro-inflammatory state, elevation oxidative stress, and reduced neuronal stimulation, appear to be influenced by sex. Therefore, aging of muscle is regulated by different mechanisms in men and women (Iannuzzi-Sucich, Prestwood & Kenny, 2002; Petersen et al., 2003).

In addition, many age-related changes in muscle mass, such as insulin resistance, pro-inflammatory state, elevation oxidative stress, and reduced neuronal stimulation, appear to be influenced by sex. Therefore, aging of muscle is regulated with different mechanisms in men and women.

We found that the genes involved in ribosome biogenesis, such as RPL24, RPS3, and other RPL families, were significantly upregulated with aging in old men.

Ribosome has an essential role in the regulation of cell proliferation and growth and homeostasis in organisms. Therefore, muscle hypertrophy requires an increase in ribosome biogenesis in smooth and skeletal muscle mass. On the other hand, mTOR is a master regulator of ribosome biogenesis by promoting the translation of the mRNAs and the transcription of rRNAs (Chaillou, Kirby & Mccarthy, 2014). Some studies have revealed that the ability of skeletal muscle to hypertrophy in response to anabolic stimulation of protein synthesis is blunted in older individuals (Hwee & Bodine, 2009; Kirby et al., 2015). Recently, Kirby et al. (2015) found that despite the ribosome protein gene expression being higher in the aged mice group, ribosome biogenesis was significantly impaired at the level of ribosomal DNA (rDNA) transcription–it had been mediated by RNA polymerase I in aged skeletal muscle. They also revealed that the greatest aged-related differences in the gene expression were RPL24 and especially RPL11. These results are consistent with another study that found that increases in muscle mass were negatively correlated with the expression of ribosomal genes (Fry et al., 2011). Therefore, our results and these reports show that the blunted hypertrophic response in old muscle was primarily the attenuated translational capacity at the level of rDNA transcription more than changes in the gene expression.

In women, our findings have shown that the expression of genes involved in lipid storage, such as, PLIN1, FABP4, and LPL, were upregulated in older individuals.

Our results might suggest developing ectopic lipid infiltration in muscle, and therefore, it may be directly linked with insulin resistance in older people. A dramatic increase in PLIN1 was observed in older women when compared to young women. Age-related loss of muscle mass depends on the decrease in muscle quality and accumulation of inter-muscular adipose tissue (IMAT). Fat accumulation could occur as intra-muscular triglycerides (IMTG) deposition in lipid droplets, which are associated with the perilipin family (Bickel, Tansey & Welte, 2009). However, previous studies found that perilipin is a protein that coats lipid droplets in adipocytes and acts as a protective factor from lipolysis (Kimmel et al., 2010; Shepherd et al., 2012). Conte et al. (2013) showed that an increased PLIN expression during aging is linked to fatty acid storage rather than utilization. It is known that fat accumulation is linked with a high flow of lipid intermediates—this causes the upregulation of peroxisome proliferator-activated receptors (PPARs) and an increased formation of oxidized lipid and Reactive Oxygen Species (ROS), thus triggering mitochondrial dysfunction and p53 activation that eventually lead to skeletal muscle atrophy in aging people (Wang & Sztalryd, 2011).

Another most upregulated DEG was fatty acid-binding protein 4 (FABP4), which is expressed in adipose tissue, heart, and skeletal muscle. It plays an important role in the development of insulin resistance and metabolic disorders. FABP4 has been shown to be released from adipocytes in a non-classical pathway associated with lipolysis, possibly acting as an adipokine (Furuhashi et al., 2014; Iso et al., 2013).

Consistent with the upregulation of lipid depositions genes in the muscle of older people, the genes involved in extracellular matrix (ECM) remodeling were also expressed at higher levels in older women than in young women (Butikofer et al., 2011; Kulakowski, Parker & Personius, 2011). The ECM provides a framework for the transmission of force and maintains a suitable environment for cellular functions. Disruption of the balance between the production of active enzymes and their inhibition may result in diseases associated with uncontrolled ECM turnover, inflammation, cell growth, and migration, such as arthritis, cardiovascular disease, cancer, and neurological disorders. The tissue inhibitors of metallo-proteinases (TIMPs) such as TIMP4, are endogenous inhibitors of these metallo-proteinases and are consequently important regulators of ECM turnover, tissue remodeling, and cellular behavior (Brew & Nagase, 2010)*.* These genes might play a role in the decline of anabolic response and insulin activity in older skeletal muscle.

Studies showed that IMATs are not always related to obesity and that healthy non-obese women can store about 60% more lipids than men in the skeletal muscle mass. Different lines of evidence have indicated that lipid accumulation inside muscle cells leads to insulin resistance (Hoeg et al., 2009). Perhaps it is the reason that many diseases related to insulin resistance, such as Type 2 diabetes, metabolic syndrome, and obesity, are higher among women.

KEGG pathway enrichment analysis showed that ribosome biogenesis was the most significantly enriched pathway for the identified DEGs in men. As mentioned, ribosome biogenesis is a central mechanism to regulate protein synthesis and control skeletal muscle size in response to anabolic and catabolic stimulation.

The most well-known cellular proteolytic system is the ubiquitinproteasome pathway (UPP), which is responsible for proteolysis (Rock et al., 1994). This is a system where proteins meant for destruction are enzymatically tagged with the polypeptide ubiquitin via E3 ubiquitin ligases. Muscle wasting is characterized by increased protein degradation via the UPP, enlarged ubiquitin conjugation to muscle proteins, and upregulation of ubiquitinprotein ligases such as CUL3 (Lecker, Goldberg & Mitch, 2006).

The peroxisome proliferator-activated receptor (PPAR) pathway is also a highly enriched pathway in women. It is a ligand-activated transcription factor with critical roles in the regulation of lipid catabolism, glucose homeostasis, and inflammation. In addition, there is strong evidence that PPAR has a role in cycle control, differentiation, and apoptosis. Also, the PPAR regulatory pathway plays an essential role in the regulation of diverse biologic processes in metabolic disorders such as diabetes, hypertension, and cardiovascular diseases (Muller, Rieck & Muller-Brusselbach, 2008).

Moreover, the KEGG pathway analysis shows that the adipocytokine signaling pathway is another highly enriched pathway in women. This pathway defines the signaling cascades arising from the adipocytokines that have been associated with insulin resistance/sensitivity and inflammation.

In our results, the expression of adiponectin gene increases in older women. Although adiponectin increases insulin sensitivity in various tissues (Zou & Shao, 2008), lipid toxicity and oxidative stress in skeletal muscle could induce the overexpression of ADIPOQ, which might potentially work as a cellular protective mechanism (Delaigle et al., 2006). Therefore, the upregulation of ADIPOQ in our study may suggest a higher propensity to develop oxidative stress and lipid storage in the muscle of elderly people.

Although hub genes and pathways are different in men and women, NTRK1, CUL3, and P53 are three significant hub proteins in PPI networks in both genders.

The common ligands of TRK receptors, a family of tyrosine kinases that regulates synaptic strength and plasticity in the nervous system, are neurotrophins. Neurotrophins are a family of growth factors critical to the functioning of the nervous system. The activation of TRK receptors by neurotrophin binding may activate the signal cascades, thereby promoting survival and other functional regulation of cells. The neurotrophic factors expressed in skeletal muscle are essential for motoneuron survival and muscle fiber innervation during development (Sakuma & Yamaguchi, 2011).

The Kelch family is considered as regulators of the processes of proliferation and/or differentiation of skeletal muscle development and function. Essentially, many Kelch proteins act as substrate-specific adaptors for Cullin E3 ubiquitin ligase (Cul3), an important component of the ubiquitin–proteasome system that regulates proteolysis (Gupta & Beggs, 2014).

Another hub protein in the PPI network was P53. It is recognized as a central regulator of cell cycle and apoptosis. During aging, P53 has been proposed to regulate both homeostasis and atrophy of skeletal muscle (sarcopenia). However, the exact molecular function of P53 remains to be clearly defined. A number of stress signals, such as oxidative stress, activate P53 and bind to the peroxisome proliferator-activated receptor gamma co-activator 1-alpha (PGC-1α) promoter for regulating an antioxidant response essential for skeletal muscle homeostasis (Aquilano et al., 2013). The nuclear S-nitrosylation of P53 significantly declines in skeletal muscle during aging, thus leading to an impairment of the homeostasis of skeletal muscle. Therefore, it could be a contributing factor of sarcopenia conditions and other skeletal muscle pathologies associated with oxidative stress (Baldelli & Ciriolo, 2016).

**Figure 2:**
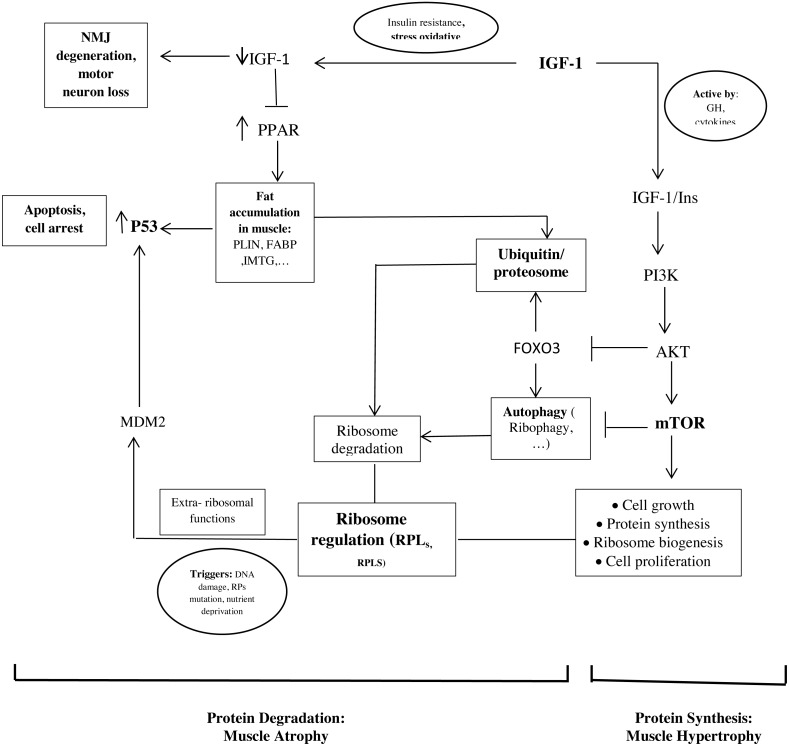
Schematic representations of the identified hub genes and proteins of muscle development, according to our results.

The important point of our study is that the hub proteins derived from the PPI network and the hub genes are interconnected (Fig. 2).

As mentioned, ribosome biogenesis genes were hub genes with the differential expression between old and young men. Recent studies found that RPs have additional extra-ribosomal functions, independent of protein biosynthesis, for the regulation of diverse cellular processes. Some triggers such as DNA damage, RP mutations, and nutrient deprivation would know as ribosomal stress to cause the release of RPs from the nucleus. Next, some RPs regulate apoptosis, cell cycle arrest, cell proliferation, neoplastic transformation, and cell migration and invasion through the activation of P53 (Xu, Xiong & Sun, 2016; Zhang & Lu, 2009). In our study, it seems that the upregulation of RPL24, RPLS3, and other RPs in the muscle of old men causes the activation of P53 and leads to cell cycle arrest and apoptosis.

In addition, the proteasome seems to play a crucial role in regulating the RP turnover through ubiquitination. It has been shown that members of the cullin family, such as NEDD8 (neural-precursor-cell-expressed developmentally downregulated 8) can have an opposite effect on cell proliferation and survival of ribosome (Caldarola et al., 2009; Xirodimas et al., 2008). Also, another mechanism of ribosome degradation is a selective autophagy called ribiophagy. For doing ribiophagy, ribosomes need to be ubiquitinated to recognize authophagy membranes (Caldarola et al., 2009).

Therefore, these mechanisms could be involved in the regulation between proliferation and cell cycle arrest by balancing the ribosome biogenesis and activating P53 or the proteolytic factors.

Our results show that genes involved in lipid storage are hub genes. NTRK1, Cullin 3 (CUL3), and P53 have been identified as hub proteins in older women. Some studies found that the association between these genes and proteins could be involved to develop old muscle mass.

In our study, we found P53 in the PPI network and the genes involved in lipid storage were the important factors in older women (Fig. 2). Another study showed that a strong correlation between the amount of P53 and the PLIN expression in aged muscle (Conte et al., 2013). P53 is known to play a role in regulating muscle atrophy. It seems that P53 activation through the PPAR pathway, possibly triggered by the accumulation of PLINs and excessive fatty acids as toxic lipid intermediates, decrease the muscle mass in older people (Wang et al., 2009). In additional, insulin is an anti-proteolytic factor, but proteolysis increases t in insulin resistance states such as accumulation of PLINs and excessive fatty acids in muscle (Conte et al., 2013; Wilkes et al., 2009).

The present results should be interpreted within the context of strengths and potential limitations. To the best of our knowledge, this is the first report using an integrated approach to identify DEGs in muscle mass between old and young individuals in both genders. We also tried to choose the most commonly used software and algorithms for structural analysis. The DEGs in the current study have been predicted without experimental evidence. Therefore, future research would be needed to prove the results of this study.

## Conclusion

By integrating the analysis of multiple gene expression profiles, we have proposed that the ribosome biogenesis genes and those involved in lipid storage would be promising markers for sarcopenia in men and women, respectively. According to PPI network analysis, neurotrophic factors expressed in skeletal muscle are essential for motoneuron survival and muscle fiber innervation during development. Cullin E3 ubiquitin ligase (Cul3) is an important component of the ubiquitin–proteasome system which regulates the proteolysis. P53 is also recognized as a central regulator of the cell cycle and apoptosis. These hub proteins might be involved in aging muscle and sarcopenia. The important point of our study is that hub proteins and hub genes are interconnected. These results could be considered meaningful for future diagnosis and therapy of sarcopenia.

##  Supplemental Information

10.7717/peerj.5239/supp-1 Table S1 The full list of the DEGs (Differentially Expressed Genes) of young and old skeletal muscle samples in both genders Raw data

10.7717/peerj.5239/supp-2 Table S2 DEGs present among top hub nodes of two gene expression data were selected ( GSE38718 and GSE25941) based on the GeneMANIA reconstructed network Full hub genes

## Additional Information and Declarations

### Data Availability

The following information was supplied regarding data availability: The full list of the DEGs is provided in Table S1.
