# Supplementary material for: Identification of candidate genes and proteins in aging skeletal muscle (sarcopenia) using gene expression and structural analysis
Source: PeerJ. 2018 Sep 5;6:e5239. doi: 10.7717/peerj.5239 (PMC6129146; doi:10.7717/peerj.5239)
Supplement: Table S2 — Full hub genes [file peerj-06-5239-s002.docx]

**Table S2.** DEGs present among top hub nodes of two gene expression data were selected (GSE38718 and GSE25941) based on the GeneMANIA reconstructed network

| **Men** | | | | | | **Women** | | | | | |
| --- | --- | --- | --- | --- | --- | --- | --- | --- | --- | --- | --- |
| **Gene symbol** | **Gene name** | **Fold change** | **P value** | **FDR** | **Degree** | **Gene symbol** | **Gene name** | **Fold change** | **P value** | **FDR** | **Degree** |
| RPL24 | *Ribosomal Protein L24* | 3.660 | 9.37E-15 | 1.39E-14 | 117 | PLIN1 | *Perilipin 1* | 6.480 | 3.37E-06 | 1.50E-03 | 39 |
| RPS3 | *Ribosomal Protein S3* | 4.819 | 5.66E-12 | 4.70E-12 | 108 | GPC3 | *Glypican 3* | 3.863 | 7.93E-06 | 2.73E-03 | 33 |
| RPL6 | *Ribosomal Protein L6* | 3.589 | 1.18E-15 | 2.18E-15 | 107 | PRL | *Prolactin* | 4.090 | 1.34E-06 | 8.10E-04 | 32 |
| RPS5 | *Ribosomal Protein S5* | 3.859 | 3.80E-10 | 2.29E-10 | 106 | PCK1 | *Phosphoenolpyruvate Carboxykinase 1* | -13.111 | 1.24E-04 | 1.30E-02 | 30 |
| PSMA3 | *Proteasome Subunit Alpha3* | 4.941 | 1.04E-23 | 3.54E-22 | 105 | ADIPOQ | *Adiponectin* | 23.072 | 2.38E-05 | 4.98E-03 | 29 |
| RPL11 | *Ribosomal Protein L11* | 3.715 | 2.53E-14 | 3.42E-14 | 103 | MNDA | *Myeloid Cell Nuclear Differentiation Antigen* | 2.305 | 3.78E-02 | 1.54E-01 | 32 |
| RPS16 | *Ribosomal Protein S16* | 3.797 | 3.91E-21 | 4.12E-20 | 101 | S100A8 | *S100 Calcium Binding Protein A8* | 3.420 | 1.80E-02 | 1.18E-01 | 30 |
| PSMA1 | *Proteasome Subunit Alpha1* | 4.607 | 6.61E-24 | 2.43E-22 | 98 | ITGB6 | *Integrin Subunit Beta 6* | -2.113 | 2.79E-03 | 5.43E-02 | 27 |
| RPL12 | *Ribosomal Protein L12* | 3.775 | 4.64E-21 | 4.79E-20 | 98 | PROK2 | *Prokineticin 2* | 2.784 | 1.40E-02 | 1.07E-01 | 26 |
| PSMB1 | *Proteasome Subunit Beta1* | 5.154 | 1.72E-26 | 3.21E-24 | 96 |  |  |  |  |  |  |
| PSMA4 | *Proteasome Subunit Alpha4* | 5.515 | 1.66E-16 | 3.81E-16 | 96 |  |  |  |  |  |  |
| RPL27 | *Ribosomal Protein L27* | 3.253 | 3.45E-24 | 1.43E-22 | 94 |  |  |  |  |  |  |
| SERPINE2 | *Serpin Family Member E2* | 4.057 | 2.09E-09 | 1.12E-09 | 12 |  |  |  |  |  |  |
| SERPINB2 | *Serpin Family Member B2* | 2.269 | 4.76E-02 | 8.52E-03 | 12 |  |  |  |  |  |  |
| SERPINE1 | *Serpin Family Member E 1* | 2.743 | 8.86E-05 | 2.44E-05 | 11 |  |  |  |  |  |  |
| ZMIZ1 | *Zinc Finger MIZ-Type Containing 1* | 5.352 | 3.08E-20 | 2.20E-19 | 11 |  |  |  |  |  |  |
| SERPINB4 | *Serpin Family Member B4* | 2.155 | 2.51E-02 | 4.70E-03 | 11 |  |  |  |  |  |  |
| PIAS2 | Protein Inhibitor Of Activated STAT 2 | 2.124 | 2.15E-16 | 4.78E-16 | 10 |  |  |  |  |  |  |
| SERPINF1 | Serpin Family Member F1 | 3.952 | 1.99E-21 | 2.32E-20 | 9 |  |  |  |  |  |  |
| SERPINA1 | Serpin Family Member A1 | 2.333 | 6.30E-03 | 1.31E-03 | 9 |  |  |  |  |  |  |
| PIAS4 | Protein Inhibitor Of Activated STAT 4 | 3.360 | 6.77E-04 | 1.64E-04 | 8 |  |  |  |  |  |  |
